# Clinical Prognostic Factors for Patients With Esophageal Cancer Treated With Definitive Chemoradiotherapy

**DOI:** 10.7759/cureus.18894

**Published:** 2021-10-19

**Authors:** Sergio L Favareto, Cecilia F Sousa, Pedro J Pinto, Henderson Ramos, Michael J Chen, Douglas G Castro, Maria L Silva, Guilherme Gondim, Antonio Cassio A Pellizzon, Ricardo C Fogaroli

**Affiliations:** 1 Radiation Oncology, AC Camargo Cancer Center, São Paulo, BRA

**Keywords:** radical treatment, overall survival, progression-free survival, chemoradiotherapy, esophageal cancer

## Abstract

Background

Treatment with definitive chemoradiotherapy (CRT) is the best option for patients with locally advanced esophageal tumors considered unresectable or for patients without clinical conditions to undergo surgical treatment. Technological advances in radiotherapy in the last decades have made treatment more accurate with less toxicity, and the association with more effective systemic treatment has been gradually improving survival rates.

Aim

Evaluate clinical prognostic factors for progression-free survival (PFS) and overall survival (OS) in patients with esophageal cancer treated with definitive radiotherapy (RT) and chemotherapy (ChT).

Material and methods

The clinical records of 60 patients treated from April 2011 until December 2019 with esophageal cancer considered unresectable and/or without clinical conditions for surgery, treated with definitive CRT, were analyzed. All patients had upper digestive endoscopy (UDE) with positive biopsy, neck, chest, and abdominal CT scan, and 18F-fluorodeoxyglucose positron-emission tomography (PET-CT). Patients were followed with physical examination and CTs every three months in the first and second years and every six months from the third year of follow-up. UDE was made every three to six months after the end of the treatment or in suspicion of tumor recurrence. PET-CT was also performed in the follow-up when clinically necessary. Local and regional failure (LRF) was defined as abnormalities in the image tests within the planning target volume (PTV) and/or positive biopsy on UDE. Any other failure was defined as a distant failure (DF). PFS was defined in the record of the first tumor recurrence site and OS in the death record from the date of the start of treatment.

Results

The median age of the patients was 66 years (range: 33 to 83 years) and 46 patients (76.7%) were male. Squamous cell carcinoma (SCC) was the most frequent histological type (85%). Most patients had tumors located in the mid-thoracic esophagus (53.3%) and stage III or IV (59.9%). All patients were treated using 3D (76.7%) or intensity-modulated radiotherapy (IMRT; 23.3%). The median total dose was 50.4Gy (41.4-50.4). All patients received platinum-based ChT concomitant with RT. The most common regimen used was carboplatin and paclitaxel, with a median of five cycles. With a median follow-up of 19 months, the median PFS and OS were 10 and 20 months, respectively. LRF and DF as the first site of failure were observed in 22 (36.6%) and 26 (43.3%) patients, respectively. In the univariate analysis, tumor length lower than 2.6 cm, gross tumor volume (GTV) volume lower than 28 cm^3^, clinical tumor stages T1 and T2, clinical node stage N0, clinical prognostic stage groups I and II, and complete response to treatment, were statistically significant factors for better PFS and OS. In the multivariate analysis, the presence of clinical nodal stage N0 was related to better PFS (p=0.02).

Conclusion

Node clinical status was the most important clinical factor for PFS. Despite all the technical progress observed in radiotherapy, treatments concomitant with platinum-based chemotherapy are associated with high levels of LRF and DF. New strategies in systemic therapy and radiotherapy are necessary for improving outcomes.

## Introduction

Esophageal cancer is the eighth most frequent malignant neoplasm and the sixth greatest cause of death from cancer in the world [1]. In 2018, according to the estimates of the Global Cancer Observatory (GLOBOCAN), the numbers of new cases and deaths were 572,034 and 508,585, respectively [2].

There are two main histological subtypes that are responsible for 95% of cases: squamous cell carcinoma (SCC) and adenocarcinoma (AC) [3]. Although the incidence of AC has increased in developed countries in recent decades, SCC remains the most common histological type in many developing countries.

Surgery is considered the best curative treatment option, but when performed alone, it presents a lower survival rate than combined treatments. Several studies have shown that the combination of surgery with radiotherapy (RT) and neoadjuvant chemotherapy (ChT) results in an increased overall survival (OS) rate when compared with surgery alone [4]. However, although the association of treatments is considered the best therapeutic option, local relapse still occurs in 40% of the patients [5]. Moreover, surgery cannot be performed in patients without favorable clinical conditions or with lesions for which resection is considered unfeasible. For these patients, chemoradiotherapy (CRT) is the treatment of choice.

Better coverage of the target volume has been possible by using three-dimensional conformal RT (3D-RT). This technique also provides more protection to risk organs, greater tumor control, and fewer side effects. 3D-RT is considered the standard technique in most studies. Currently, with intensity-modulated radiotherapy (IMRT), it is possible to achieve even greater accuracy in the coverage of the target volume, with lower toxicity.

However, despite all technological advances in RT and surgery and a better understanding of the clinical and biological characteristics of the disease, esophageal cancer survival remains low.

This article analyzes a population that is highly representative of the clinical reality of esophageal cancer patients from a developing country who were treated definitively with CRT. It seeks to identify the clinical factors that influence disease-free survival (DFS) and overall survival (OS).

## Materials and methods

The present study was approved by the Research Ethics Committee of the AC Camargo Cancer Center before the collection of data from the patients' clinical records.

Patient population

This study retrospectively analyzed the clinical data of patients diagnosed with esophageal cancer who either had tumors whose resection was considered unfeasible or were considered not to be in a clinical condition to undergo surgical treatment and therefore were treated with definitive CRT in the Radiotherapy Department of the AC Camargo Cancer Center from April 2011 to December 2019. Patients with a confirmed esophageal SCC or AC diagnosis confirmed by a biopsy and who were submitted to curative treatment with CRT were included. Patients treated with CRT intended to be neoadjuvant (lower final dose) but who subsequently did not undergo surgery were also included. Patients were excluded if they did not complete the proposed treatment, received surgical treatment, had a clinical history of other neoplasms prior or simultaneous to the treatment of esophageal neoplasms, were undergoing palliative treatment, or had metastases at diagnosis. The final cohort included 60 patients.

Treatment

All patients underwent previous planning for the treatment with 3D-RT or IMRT. The final dose used ranged from 41.4 to 50.4 Gy, with a dose per fraction of 1.8 Gy/day. The gross tumor volume (GTV) included the tumor and lymph nodes considered positive by fluorine-18 fluorodeoxyglucose positron-emission tomography/computed tomography (18F-FDG PET/CT) or endoscopic ultrasound. The clinical target volume (CTV) included GTV, a 4-5-cm additional cranial-caudal esophagus margin, and a 1-cm radial margin. For tumors of the cervical or upper thoracic esophagus, the lymph nodes of the supraclavicular fossa were included in the CTV. Celiac trunk lymph nodes were also included in the CTV for tumors in the esophagogastric transition or in the distal third of the thoracic esophagus. For the planning target volume (PTV), an additional margin of 0.5-1.0 cm was applied. Patients received treatment with CRT and platinum-based ChT. The most commonly used ChT scheme was the combination of paclitaxel (50 mg/m^2^ intravenously on Day 1) and carboplatin (area under the curve (AUC) 2 intravenously on Day 1) weekly.

All patients underwent clinical review consultation and nutritional status assessment during treatment.

Follow-up

The patients were followed up, with physical examination and chest and abdomen CT every three months in the first two years of follow-up and every six months from the third year onward. Upper digestive endoscopy (UDE) was performed every three to six months after the end of the treatment during the first two years of follow-up, every six months after two years, or at any time in case of suspected recurrence. When judged clinically relevant, 18F-FDG PET/CT was also performed.

Locoregional failure (LRF) was defined as the presence of any abnormalities in imaging tests within the PTV or a positive biopsy during UDE. Any other failure was defined as a distance failure (DF). Progression-free survival (PFS) was defined as the time elapsed from the date of initiation of radiotherapy to the date of identification of the first site of tumor recurrence, and overall survival (OS) was defined as the period from the date of initiation of radiotherapy to the date of death registration.

Statistical analysis

Initially, a descriptive analysis of the variables was performed, in which the absolute (n) and relative (%) frequency distributions were presented for qualitative variables, and the main summary measures, such as mean, standard deviation, median, and minimum and maximum values, were calculated for quantitative variables.

The Kaplan-Meier estimator was considered when determining the survival curves, and groups were compared for their survival rates using the log-rank test. The Cox regression model was used to perform both simple and multiple hazard ratio (HR) evaluations. Proportional hazard assumptions were verified through Schoenfeld residuals.

A 5% significance level was adopted, and statistical analyses were performed using SPSS version 25 (IBM Corp., Armonk, NY) and free R software version 3.6.2 (R Foundation for Statistical Computing, Vienna, Austria).

## Results

Data on patients, tumors, and treatments are furnished in Table 1.

**Table 1:** Overview: patients characteristics (n=60) EGT: esophagogastric transition tumor, RT: radiotherapy, ChT: chemotherapy, Gy: Gray *1- KPS (Karnofsky performance status) score *2- Clinical tumor (cT) and clinical lymph-node (cN) stage was assessed by means of endoscopic ultrasound, computed tomography or 18F-fluorodeoxyglucose positron-emission tomography. The tumor stage, nodal stage, and clinical prognostic groups were classified according to the International Union against Cancer (UICC) tumor—node—metastasis (TNM) classification, eighth edition, 2017. *3- Tumor length and location were determined by means of endoscopy.

Characteristics		No. (range, %)
age (years)	median	66 (33-83)
sex	male; female	46 (76.7); 14 (23.3)
KPS*^1^	median	80 (50-90)
clinical tumor (T) stage*^2^	T1; T2; T3; T4a; T4b	4 (6.7); 12 (20.0); 32 (53.3); 1 (1.7); 11 (18.3)
clinical nodal (N) stage*^2^	N0; N1; N2	23 (38.3); 19 (31.7); 18 (30.0)
clinical prognostic group *^2^	I; II; III; IVA	4 (6.7); 20 (33.3); 23 (38.3); 13 (21.7)
histologic grade	GX; G1; G2; G3	5 (8.3); 7 (11.7); 28 (46.7); 20 (33.3)
histology	squamous cell carcinoma; adenocarcinoma	51 (85); 9 (15)
tumor length*^3^ (cm)	median	5 (1-9.9)
Tumor site	cervical; upper thoracic; middle thoracic; low thoracic; EGT	9 (15); 13 (21.7); 26 (43.3); 8 (13.3); 4 (6.7)
RT technique	3D; IMRT	46 (76.7); 14 (23.3)
RT dose (Gy)	median	50.4 (41.4-50.4)
GTV volume (cm³)	median	49 (3.92-373.47)
number of ChT cycles	median	5 (2-7)
complete response	yes; no	35 (58.3), 25 (41.7)

Survival

**Figure 1:**
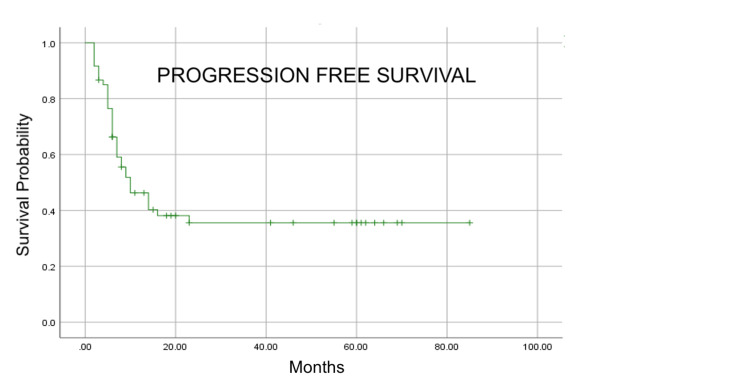
Kaplan–Meier estimation of progression-free survival

**Figure 2:**
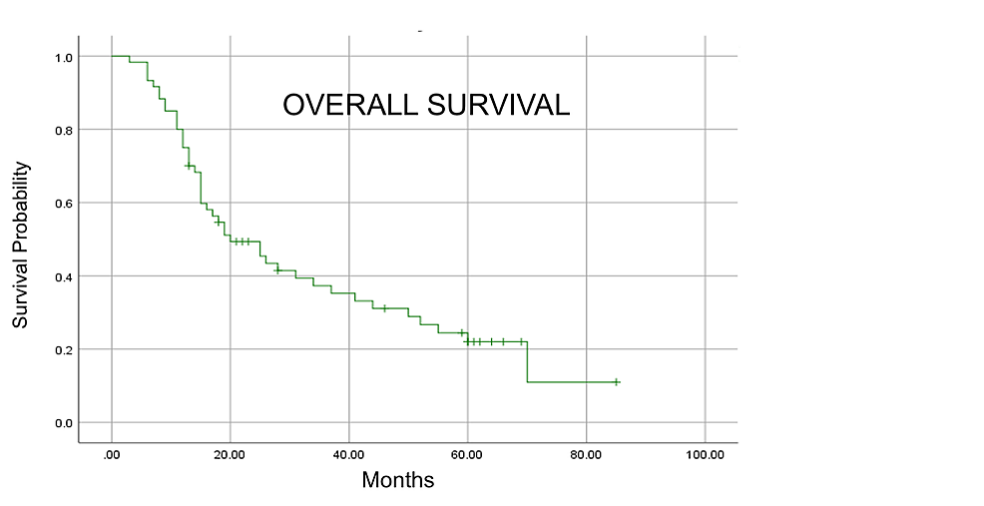
Kaplan–Meier estimation of overall survival

With a median follow-up of 19 months, median PFS and median OS were 10 and 20 months, respectively (Figure 1 and Figure 2).

No statistically significant relationship was found with regard to the variables gender, age, histological type, tumor location, histological grade, treatment technique, the dose of radiotherapy, and the number of chemotherapy cycles and either OS or PFS.

Disease-free survival

**Figure 3:**
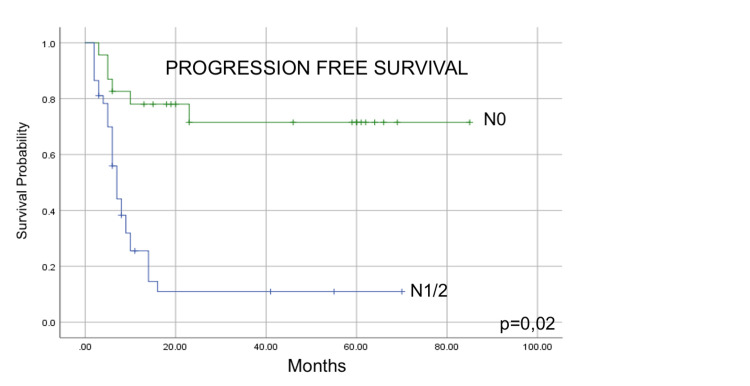
Kaplan-Meier estimation of progression-free survival (PFS) for patients with clinical node stage N0 vs. N1/2 (p=0.02)

Table 2 shows the variables that influenced DFS in the univariate and multivariate analyses. In the univariate analysis, the variables that stood out as predictors of better DFS rates were prognostic groups I and II (p = 0.001) and complete response to treatment, with median DFS values much higher (23 months vs. 7 months; p = 0.003) than those with incomplete response and absence of positive lymph nodes (p < 0.001). In the multivariate analysis, the lymph node status remained a statistically significant predictive factor (p = 0.02) (Figure 3).

**Table 2:** Hazard ratios in univariate and multivariate analysis of PFS GTV: gross tumor volume; HR: hazard ratio; PFS: progression-free survival

parameter	n	univariate analysis		multivariate analysis	
		HR (95% CI)	p-value	HR (95% CI)	p-value
tumor length		3.15 (1.10-8.97)	p=0.03	1.89 (0.62-5.75)	p=0.25
≤2.6 cm	12				
>2.6 cm	48				
GTV		4.18 (1.45-12.03)	p=0.008	0.91 (0.20-4.05)	p=0.90
≤28 cm^3^	14				
>28 cm³	46				
clinical tumor (T) stage		2.49 (1.03-6.01)	p=0.04	1.62 (0.37-7.08)	p=0.52
T1 and T2	16				
T3 and T4	44				
clinical node (N) stage		5.71 (2.32-14.02)	p<0.001	6.23 (1.26-30.81)	p=0.02
N0	23				
N1/2	37				
clinical prognostic group		3.87 (1.73-8.62)	p=0.001	0.84 (0.11-6.08)	p=0.86
I and II	24				
III and IV	36				
complete response		2.82 (1.41-5.61)	p=0.003	1.26 (0.60-2.62)	p=0.53
yes	35				
no	25				

Overall survival

With regard to OS, as observed for PFS, the following variables were associated with lower OS in the univariate analysis: tumor length >2.6 cm, GTV >28 cm3, stage T3 and T4 tumors, presence of positive lymph nodes, clinical prognostic groups III and IV, and absence of complete response after completion of treatment. In the multivariate analysis, complete response was the best-correlated variable, with the best median OS rate (44 months vs. 15 months), although it was not statistically significant (p = 0.09) (Table 3).

**Table 3:** Hazard ratios in univariate and multivariate analysis of OS GTV: gross tumor volume; HR: hazard ratio; OS: overall survival

parameter	n	univariate analysis		multivariate analysis	
		HR (95% CI)	p-value	HR (95% CI)	p-value
Tumor length		2.56 (1.07-6.10)	p=0.03	1.45 (0.58-3.61)	p=0.42
≤2.6 cm	12				
>2.6 cm	48				
GTV		3.94 (1.79-8.64)	p=0.001	1.58 (0.43-5.80)	p=0.48
≤28 cm^3^	14				
>28 cm³	46				
clinical tumor (T) stage		2.96 (1.30-6.66)	p=0.009	1.98 (0.49-7.94)	p=0.33
T1 and T2	16				
T3 and T4	44				
clinical node (N) stage		3.88 (1.83-8.21)	p<0.001	2.50 (0.53-11.8)	p=0.24
N0	23				
N1	37				
clinical prognostic group		3.05 (1.52-6.12)	p=0.002	0.56 (0.11-2.90)	p=0.49
I and II	24				
III and IV	36				
complete response		3.57 (1.92-6.63)	p<0.001	1.84 (0.89-3.78)	p=0.09
yes	35				
no	25				

LRF was detected in 22 patients (36.7%), all confirmed by biopsy. DF was observed in 26 patients (43.3%), and 77% of them had tumors of prognostic groups III and IV. The main sites of metastases were the lung (72.7%), liver (29.2%), and bones (25%).

All patients had either odynophagia or dysphagia or both as side effects during treatment, and in 26 patients (43.3%), these symptoms were classified as Grade 3 according to the Radiation Therapy Oncology Group (RTOG) classification [6].

## Discussion

In this study, a retrospective analysis of patients with esophageal cancer treated with CRT was conducted. Although there are limitations inherent to this modality of study, it can offer a faithful picture of the everyday reality of radiotherapy centers. Moreover, it has practical significance in identifying clinical aspects related to the success or failure of specific treatment modalities in patients whose clinical condition often does not meet the criteria for inclusion in phase II and III studies. The AC Camargo Cancer Center treats patients with public and private health insurance, and SCC histology and clinical stages III and IV were found in 85% and 60% of cases, respectively, a scenario still common in developing countries.

Most studies that analyzed the results of definitive CRT treatments showed lower OS results than those obtained with neoadjuvant treatment followed by surgery, and this modality is considered the preferred treatment for patients with favorable clinical conditions and tumors that can be resected [3-5]. Although some studies did not show survival benefits with the addition of surgery in relation to treatment with definitive CRT, they used more toxic chemotherapy regimens and presented higher surgical mortality compared to current studies [7-8].

When surgical treatment is not possible, definitive treatment with CRT is the standard approach, offering better results than those obtained with radiotherapy alone. The classical RTOG 85-01 study involving 192 patients showed higher survival rates for RT (50 Gy) associated with ChT with cisplatin and 5FU than for RT alone [9]. In a study by Haefner et al., CRT proved to be an independent prognostic factor in the multivariate analysis for better DFS and OS [10]. In recent decades, several CRT schemes have been tested. Table 4 presents the results of these studies in comparison with those of the present study.

**Table 4:** Trials evaluating definitive chemoradiation in esophageal cancer AD: adenocarcinoma; Cetux: cetuximab; CF: cisplatin and 5-fluorouracil; CX: cisplatin and capecitabine; DCF: docetaxel, cisplatin, and 5-fluorouracil; F: 5-fluorouracil; FEP: 5-fluorouracil, etoposide, and cisplatin; Folfox: 5-fluorouracil and oxaliplatin; Iri: irinotecan; PTX: paclitaxel; Carbo: carboplatin; NR: not reported; PFS: progression-free survival; OS: overall survival; SCC: squamous cell carcinoma; ChT: chemotherapy; RT: radiotherapy; Gy: Gray; ACCCC: AC Camargo Cancer Center

author	N	histology (%)	ChT	RT dose	PFS	OS
				(Gy)	(months)	(median)
Conroy T [11]	267	SCC: 85; AD:14	Folfox vs.CF	50	9.7m vs. 9.4m	20m vs.17.5m
							Crosby T [12]	258	SCC: 73; AD:25	CX +/- Cetux	50	16m vs.21.6m	22m vs. 25 m
Tomblyn MT [13]	21	SCC: 50; AD:50	Cis/Iri/Cetux	50.4	6.4m	11.2m
Bedenne L [7]	130	SCC: 90; AD:10	CF	66	NR	19.3m
Stahl M [10]	86	SCC: 100	FEP	65	NR	14.9m
Minsky BD [14]	218	SCC: 86; AD:14	CF	64.8 vs. 50.4	NR	13 vs. 18m
Higuchi K [15]	42	SCC:83; AD::17	DCF	61.2-50.4	NR	29m
Kato K [16]	51	SCC:100	CF	50.4	NR	NR
Tang HR [17]	76	SCC: 100	Cis/PTX	68.4 or 61.2	NR	28.5m
Teoh AY [18]	81	SCC:100	CF	50–60	NR	NR
ACCCC	60	SCC:85; AD:15	Carbo/PTX	50.4	10m	20m

The present study obtained a median PFS of 10 months and a median OS of 20 months, which are compatible with the findings of other studies [7,11,14]. Some factors, such as the gender of the patients, the tumor site, and the type of histology in DFS or OS, did not have a statistically significant influence.

There was a predominance of patients with tumors of SCC histology (85%), and it was not possible to identify any influence of the type of histology on DFS or OS. This subject is a point of controversy in the literature. When analyzing 1,059 patients undergoing surgery, Siewert et al. [19]. found that AC histology presented a more favorable prognosis than SCC. However, other series analyzing patients undergoing treatment with neoadjuvant or definitive CRT did not confirm this finding [5,8,13,20]. On the contrary, the CROSS study analyzing patients treated with neoadjuvant therapy showed a higher complete response rate (p = 0.008) and greater survival benefit for SCC histology [4].

The following variables exerted their influence on DFS and OS in the present study:

*1) Positive lymph nodes:* The presence of positive regional lymph nodes was the only variable in our study that remained an independent factor in the multivariate analysis for PFS (p = 0.02; Figure 3). This finding is frequently mentioned in studies that analyze treatments with both neoadjuvant and definitive purposes [20-22]. Mariette et al. [20] analyzed 536 patients undergoing esophagectomy, 51.5% of whom received neoadjuvant CRT. After a median follow-up of 50 months, the five-year survival rate was 47% for the entire population and was significantly less for patients with more than four positive lymph nodes (8% vs. 53%; p < 0.001) or with a proportion of positive lymph nodes in relation to total resected lymph nodes of >0.2 (22% vs. 54%; p < 0.001). In the adjusted analysis, both factors were the only predictors of worse prognosis for patients undergoing neoadjuvant treatment. The influence of the extent of lymph node involvement was analyzed by Huang et al. [22] in a study of definitive CRT patients with cT4b tumors of predominant SCC histology (94%). Clinical stage cN0 or cN1 was identified as an independent prognostic variable for better survival in relation to stage cN2 or cN3 (p = 0.014).

*2) Staging:* In the present study, patients with early (I and II) and advanced (III and IV) stage tumors had a median OS of 55 and 15 months, respectively. Different inclusion criteria employed in various studies may explain the differences in OS. The present study obtained a median OS of 20 months, which can be explained by the inclusion of patients from prognostic groups I and II (40%). This influence of advanced stages on OS has been investigated by other authors [10,18]. Haefner et al. [10] obtained a median OS of 15 months, which can be explained by the inclusion of patients with metastases (22.7%). Bedenne et al. [7], on the other hand, in their study on the impact of surgery on survival in relation to CRT alone, obtained a total median survival of 19 months and a two-year OS of 39.8%, excluding patients with T4 or M1 tumors and those who did not respond to the initial induction CRT.

*3) Tumor length and volume: *Tumor length and GTV can be measured easily through endoscopy and during radiotherapy planning. These parameters have been shown to be prognostic factors in several studies. Wang et al. established that the five-year survival rates were 77.3%, 48.1%, 38.5%, and 23.3% for tumor lengths of 1 cm, 2 cm, 3 cm, and >3 cm, respectively (p < 0.001), and the latter parameter remained a statistically significant prognostic factor in the multivariate analysis [23]. Another study was conducted using data from the National Cancer Institute Surveillance, Epidemiology, and End Results and demonstrated that a tumor length >3 cm was a predictor of mortality [24]. The importance of this variable was recognized by van Hagen et al. whose CROSS study limited the inclusion of patients to those with tumors ≤8 cm in length [5]. The findings of the present study confirmed these data, and the median OS for patients with tumors ≤2.6 cm and >2.6 cm were 55 and 18 months, respectively. GTV, a variable directly linked to clinical staging, was also identified in the univariate analysis as a predictor of better DFS (p = 0.008) and OS (p = 0.001).

*4) Response pattern: *In the present study, 35 patients (54.7%) had a complete response, with a significant impact on median OS (44 vs. 15 months). This value is higher than that found by other researchers and can be explained by the inclusion of 24 patients (40%) belonging to prognostic groups I and II [5,13]. The influence of complete response was also identified by Huang et al. in the treatment of patients with T4b tumors. The overall treatment response rate was 76.9%, and a complete response was observed in 25% of the patients who had a significant median survival rate of 81.6 months [22].

LRF and DF are still very common outcomes in esophageal cancer. Several studies have been and are being conducted on RT dose escalation and the use of new systemic agents in an attempt to improve these parameters.

The present study found LRF in 36.7% of patients. Studies have tried to escalate the treatment dose, aiming at greater local control. An analysis of data from the National Cancer Database by Brower et al. discerned no difference in SG between patients receiving 50-50.4 Gy and those receiving >50.4 Gy. However, locoregional control was not analyzed in that study [25]. Other authors have found different results, showing an improvement in local control and in survival with dose escalation. In a phase I/II study, Chen et al. analyzed 46 patients with locally advanced or metastatic esophageal cancer [26]. They were treated with a dose of 50.4 Gy in 28 fractions, with an integrated 63 Gy boost in tumor and lymph node GTV, establishing this treatment regimen to be well-tolerated. Extrapolating these data, in a comparison with data from the same institution, the use of an integrated boost achieved higher local control, with HR 0.49 (p = 0.03), and higher OS, with HR 0.66 (p = 0.02).

Currently, several phase III studies are underway, such as the French CONCORDE/PRODIGE 26 trial (NCT01348217) comparing 66 Gy and 50 Gy doses combined with 5-fluorouracil and oxaliplatin (Folfox) ChT [27]. With the same objective, the Dutch ART DECO study is evaluating the dose increase in patients with non-operable esophageal cancer ​(50.4 vs. 61 Gy) with an increased dose per fraction and using a concomitant boost, associated with ChT with carboplatin and paclitaxel.

Proton-beam RT (PBT) was evaluated in a retrospective study by Xi et al. When compared with IMRT, PBT was associated with better OS and PFS [28]. A number of randomized prospective studies are underway comparing PBT and IMRT (NCT03801876, NCT01512589).

Obviously, although local control is an important objective, DF remains a frequent finding. In the present study, 40% of the patients evolved with DF. A combination of carboplatin and paclitaxel was used as the ChT regimen in the present study. This regimen is recommended by the National Comprehensive Cancer Network as one of the options indicated for association with RT.

The phase II RTOG 0113 study attempted to boost the systemic treatment by using three drugs. Patients with tumors that could not be resected and those who did not wish or had no clinical condition to undergo surgery were randomized into two groups [29]. Group A received induction ChT with 5-fluorouracil, cisplatin, and paclitaxel, followed by 5-fluorouracil and paclitaxel with RT (50.4 Gy). Group B received induction CT with paclitaxel plus cisplatin, followed by the same ChT scheme with RT (50.4 Gy). A total of 72 patients were evaluated (group A, n = 37; group B, n = 35). The median survival was 28.7 months for group A patients and 14.9 months for group B. However, toxicity was high in both groups, with frequent grade 3 toxicity (group A = 54%, group B = 43%) and grade 4 toxicity (group A = 27%, group B = 40%). Treatment-related death occurred in 3% of group A patients and 6% of group B patients. Other Asian authors, in studies with the same objective and limited numbers of patients, obtained median survival rates >28 months, with RT dose escalation associated with ChT regimens using two or three drugs, but with significant toxicity [15-16].

The expression of the epidermal growth factor receptor is associated with a worse prognosis [30]. However, contrary to the satisfactory results obtained with the association of RT and cetuximab in the treatment of head and neck tumors, the same was not observed for esophageal tumors. In a multicenter phase II/III study (SCOPE1), 258 patients with non-metastatic stage I-III esophagus carcinoma were randomized to receive CRT with cisplatin, capecitabine, and RT at a dose of 50 Gy, or the same treatment regimen with the addition of cetuximab. DFS at 24 weeks was lower in the cetuximab group than in the group treated with CRT alone (66.4% vs. 76.9%). The median OS was also lower in the cetuximab group (22.1 months vs. 25.4 months). Patients who received CRT plus cetuximab had more grade 3 or 4 non-hematological toxicities (79% vs. 63%; p = 0.004) [12].

## Conclusions

The treatment of esophageal cancer in advanced stages with the traditional radiotherapy regimen with a median dose of 50.4 Gy and chemotherapy based on platinum derivatives seems to have reached its effectiveness limit, with unsatisfactory DFS and OS rates. Variables that influence these results, such as the presence of positive lymph nodes, prognostic groups III and IV, and T3 and T4 tumors, have been identified both in the present study and in those by other authors. A forward step is still awaited, with new treatment schemes aimed at increasing local control and reducing distant failures, associated with reasonable toxicity levels.
